# Erratum

**DOI:** 10.1093/ehjdh/ztab098

**Published:** 2021-11-21

Upon the original publication of the below listed articles, the Data Availability statement was inadvertently omitted. This erratum has been published to address the omission and note that the statements have been instated for the following papers:

Enhanced prediction of atrial fibrillation and mortality among patients with congenital heart disease using nationwide register-based medical hospital data and neural networks

*European Heart Journal–Digital Health*, ztab065, https://doi.org/10.1093/ehjdh/ztab065

Digital transformation of major scientific meetings induced by the COVID-19 pandemic: insights from the ESC 2020 annual congress

*European Heart Journal–Digital Health*, ztab076, https://doi.org/10.1093/ehjdh/ztab076

Social media in cardiovascular medicine: a contemporary review

*European Heart Journal–Digital Health*, Volume 1, Issue 1, November 2020, Pages 10–19, https://doi.org/10.1093/ehjdh/ztaa004

Contact-free sensor signals as a new digital biomarker for cardiovascular disease: chances and challenges

*European Heart Journal–Digital Health*, Volume 1, Issue 1, November 2020, Pages 30–39, https://doi.org/10.1093/ehjdh/ztaa006

Effect of a pragmatic home-based mobile health exercise intervention after transcatheter aortic valve replacement: a randomized pilot trial

*European Heart Journal–Digital Health*, Volume 2, Issue 1, March 2021, Pages 90–103, https://doi.org/10.1093/ehjdh/ztab007

Artificial intelligence assessment for early detection of heart failure with preserved ejection fraction based on electrocardiographic features *European Heart Journal–Digital Health*, Volume 2, Issue 1, March 2021, Pages 106–116, https://doi.org/10.1093/ehjdh/ztaa015.

The effect of confounding data features on a deep learning algorithm to predict complete coronary occlusion in a retrospective observational setting

*European Heart Journal–Digital Health*, Volume 2, Issue 1, March 2021, Pages 127–134, https://doi.org/10.1093/ehjdh/ztab002

Electrocardiogram machine learning for detection of cardiovascular disease in African Americans: the Jackson Heart Study

*European Heart Journal–Digital Health*, Volume 2, Issue 1, March 2021, Pages 137–151, https://doi.org/10.1093/ehjdh/ztab003

Potential of eHealth smart technology in optimization and monitoring of heart failure treatment in adults with systemic right ventricular failure

*European Heart Journal–Digital Health*, Volume 2, Issue 2, June 2021, Pages 215–223, https://doi.org/10.1093/ehjdh/ztab028

Patient-reported outcomes in symptom-driven remote arrhythmia monitoring: evaluation of the Dutch HartWacht-telemonitoring programme

*European Heart Journal–Digital Health*, Volume 2, Issue 2, June 2021, Pages 224–230, https://doi.org/10.1093/ehjdh/ztab030

Transition of May Measurement Month to an online hypertension awareness campaign in Korea during the COVID-19 pandemic *European Heart Journal–Digital Health*, Volume 2, Issue 2, June 2021, Pages 254–258, https://doi.org/10.1093/ehjdh/ztab019.

Assessing the methodology used to study the ascending aorta haemodynamics in bicuspid aortic valve

*European Heart Journal–Digital Health*, Volume 2, Issue 2, June 2021, Pages 271–278, https://doi.org/10.1093/ehjdh/ztab022

Advanced heart sound analysis as a new prognostic marker in stable coronary artery disease

*European Heart Journal–Digital Health*, Volume 2, Issue 2, June 2021, Pages 279–289, https://doi.org/10.1093/ehjdh/ztab031

Mapping and quantification of the twitter footprint of cardiologists

*European Heart Journal–Digital Health*, Volume 2, Issue 3, September 2021, Pages 374–378, https://doi.org/10.1093/ehjdh/ztab049

Echocardiographic phenogrouping by machine learning for risk stratification in the general population

*European Heart Journal–Digital Health*, Volume 2, Issue 3, September 2021, Pages 390–400, https://doi.org/10.1093/ehjdh/ztab042

Deep learning analysis of resting electrocardiograms for the detection of myocardial dysfunction, hypertrophy, and ischaemia: a systematic review

*European Heart Journal–Digital Health*, Volume 2, Issue 3, September 2021, Pages 416–423, https://doi.org/10.1093/ehjdh/ztab048

Applications of artificial intelligence/machine learning approaches in cardiovascular medicine: a systematic review with recommendations

*European Heart Journal–Digital Health*, Volume 2, Issue 3, September 2021, Pages 424–436, https://doi.org/10.1093/ehjdh/ztab054

The discerning ear: cardiac auscultation in the era of artificial intelligence and telemedicine *European Heart Journal–Digital Health*, Volume 2, Issue 3, September 2021, Pages 456–466, https://doi.org/10.1093/ehjdh/ztab059

Kardia Mobile and ISTEL HR applicability in clinical practice: a comparison of Kardia Mobile, ISTEL HR, and standard 12-lead electrocardiogram records in 98 consecutive patients of a tertiary cardiovascular care centre

*European Heart Journal–Digital Health*, Volume 2, Issue 3, September 2021, Pages 467–476, https://doi.org/10.1093/ehjdh/ztab040

Personalized teleprehabilitation in elective cardiac surgery: a study protocol of the Digital Cardiac Counselling randomized controlled trial

*European Heart Journal–Digital Health*, Volume 2, Issue 3, September 2021, Pages 477–486, https://doi.org/10.1093/ehjdh/ztab041

